# Nonfunctioning Juxtaglomerular Cell Tumor

**DOI:** 10.1155/2013/973865

**Published:** 2013-03-27

**Authors:** Ryoko Sakata, Hiroaki Shimoyamada, Masahiro Yanagisawa, Takayuki Murakami, Kazuhide Makiyama, Noboru Nakaigawa, Yoshiaki Inayama, Kenichi Ohashi, Yoji Nagashima, Masahiro Yao, Yoshinobu Kubota

**Affiliations:** ^1^Department of Urology, Yokohama City University Graduate School of Medicine, 3-9 Fukuura, Kanazawa-ku, Yokohama 236-0004, Japan; ^2^Department of Pathology, Yokohama City University Graduate School of Medicine, 3-9 Fukuura, Kanazawa-ku, Yokohama 236-0004, Japan; ^3^Department of Pathology, Kyorin University Graduate School of Medicine, 6-20-2, Shinkawa, Mitaka, Tokyo 181-8611, Japan; ^4^Division of Anatomical and Surgical Pathology, Yokohama City University Hospital, 3-9 Fukuura, Kanazawa-ku, Yokohama 236-0004, Japan

## Abstract

The juxtaglomerular cell tumor (JGCT) is a rare renal tumor characterized by excessive renin secretion causing intractable hypertension and hypokalemia. However, asymptomatic nonfunctioning JGCT is extremely rare. Here, we report a case of nonfunctioning JGCT in a 31-year-old woman. The patient presented with a left renal tumor without hypertension or hypokalemia. Under a clinical diagnosis of renal cell carcinoma, radical nephrectomy was performed. The tumor was located in the middle portion adjacent to the renal pelvis, measuring 2 cm in size. Pathologically, the tumor was composed of cuboidal cells forming a solid arrangement, immunohistochemically positive for renin. Based on these findings, the tumor was diagnosed as JGCT. In cases with hyperreninism, preoperative diagnosis of JGCT is straightforward but difficult in nonfunctioning case. Generally, JGCT presents a benign biological behavior. Therefore, we should take nonfunctioning JGCT into the differential diagnoses for renal tumors, especially in younger patients to avoid excessive surgery.

## 1. Introduction

Juxtaglomerular cell tumor (JGCT), a neoplasm derived from the juxtaglomerular cell of the kidney, was first described by Robertoson et al. and Kihara et al. [1, 2]. Since then, approximately 100 cases have been reported in the literature. Clinically, this tumor is characterized by hypertension due to excessive renin secretion by tumor cells causing secondary hyperaldosteronism [1–3]. Generally, its preoperative diagnosis is relatively easy, because of typical presence of hypertension concomitant with hypokalemia. However, it is quite difficult in cases of asymptomatic nonfunctioning JGCT [3]. Here, we present a case of nonfunctioning JGCT, which is the fourth case reported so far to the best of our knowledge [4–6]. 

## 2. Case Report

**Figure 1:**
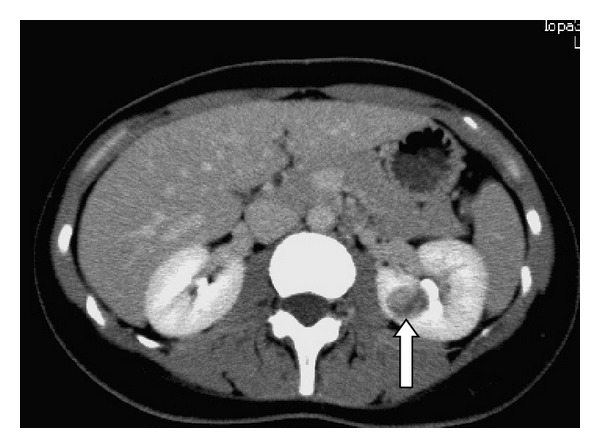
Enhanced CT showing the tumor (excretory phase) (arrow).

A 31-year-old woman was referred to our hospital with a left renal tumor incidentally detected during examination for left-side abdominal pain. She had no history of hypertension, and her blood pressure at presentation was 116/62 mmHg. All the laboratory data, including electrolyte levels, were within normal ranges. Unfortunately, preoperative plasma renin activity was not assayed. Unenhanced computed tomography (CT) revealed solitary well-circumscribed mass lesion measuring 2 cm with fine calcifications in the middle portion adjacent to the renal pelvis of the left kidney. Dynamic-enhanced CT demonstrated that the tumor was not enhanced in the corticomedullary (early) phase but enhanced in the excretory (late) phase (Figure 1). Magnetic resonance image (MRI) showed a well-defined, round mass in the middle portion of the kidney that appears isointense on T1-weighted image. The mass showed low intensity in fat saturation T2-weighted image. With a clinical diagnosis of renal cell carcinoma, the patient underwent laparoscopic radical nephrectomy. 

**Figure 2:**
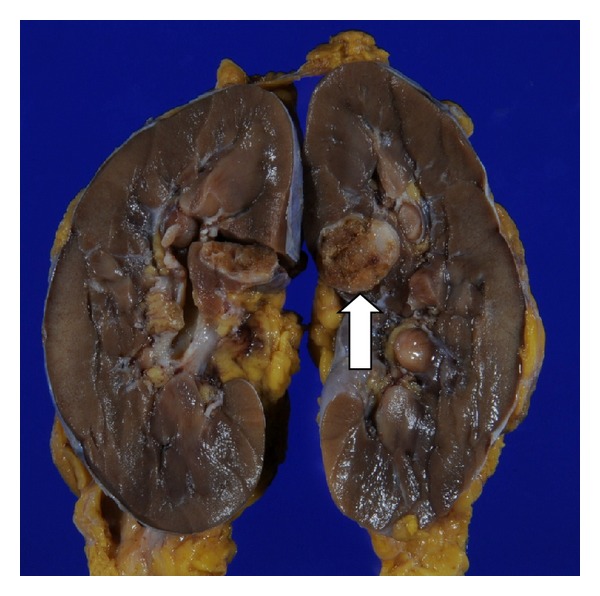
Gross findings of the resected kidney. The tumor located in the middle portion and was well demarcated from the surrounding structures. The cut surface was white to tan in color.

Grossly, the resected kidney contained an encapsulated yellowish white mass in the middle portion (Figure 2). 

**Figure 3:**
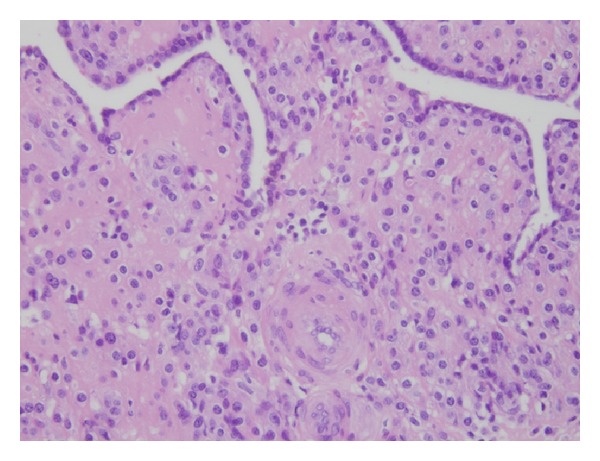
Histological findings of the tumor. The tumor was composed of solid cell sheets containing tubular architectures.

**Figure 4:**
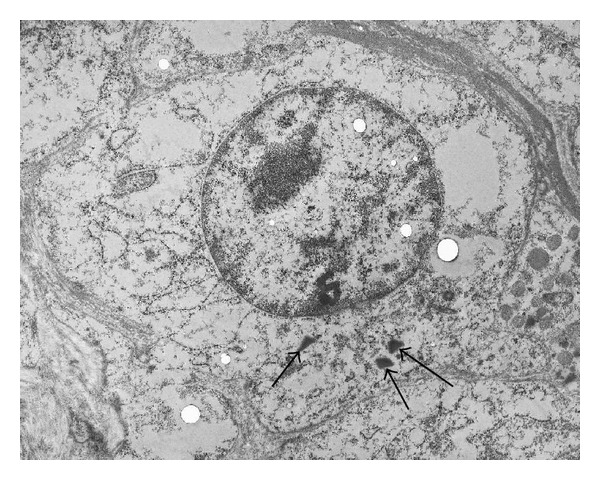
Electron micrograph of a tumor cell. Rhomboidal crystalline bodies are observed in the cytoplasm of a tumor cell (arrow).

**Figure 5:**
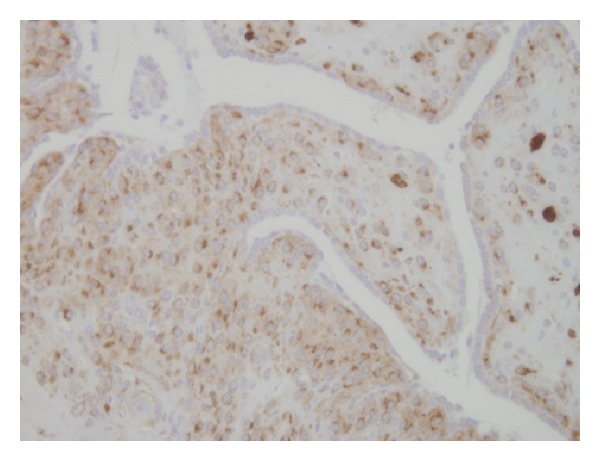
Immunohistochemical micrograph of the tumor. The majority of tumor cells were positive for renin, although the tubular lining was negative.

Histologically, the tumor was composed of densely packed cells surrounding tubular spaces with cuboidal lining. The tumor cells were uniformly small in size, polygonal in shape, possessed small round nuclei, and were clear to eosinophilic cytoplasm. Their nuclei contained fine chromatin and inconspicuous nucleoli (Figure 3). Ultrastructurally, the tumor cells contained rhomboid-shaped granules in the cytoplasm, consistent with renin protogranules (Figure 4). Immunohistochemically, the tumor cells showed strong positivity for renin (Figure 5) but were negative for cytokeratin and epithelial membrane antigen. Because epithelial markers, but not renin, were positive in the space-lining cells, the tubular architectures seemed to be derived from dilated tubules involved by the tumor. 

Based on these findings, the tumor was diagnosed as nonfunctioning JGCT. Postoperatively, the patient is doing well for more than 3 years of followup. 

## 3. Discussion

JGCT is a rare benign renal neoplasm, which mostly affects the younger females (second to third decades; mean age, 27 years old; male : female = 1 : 2). It is most frequently found during the examination for intractable hypertension. 

On plain CT, JGCT usually appears as a unilateral well-circumscribed hypoattenuating cortical mass. At dynamic CT, the tumors are not enhanced in the arterial phase despite the profuse vascularity, possibly because of renin-induced vasoconstriction. The tumors show moderate enhancement during the delayed phase [7, 8]. At MRI, JGCT may appear as a cortical-based renal mass of variable signal intensity that shows delayed peripheral enhancement on dynamic contrast-enhanced MRIs [9].

Grossly, the tumor is well circumscribed and firm in consistency, and the cut surface is yellowish to tan in color. Histologically, the tumor is composed of sheets, papillae, and trabeculae of uniform polygonal cells. Their nuclei are small with fine chromatin, and the cytoplasm is granular. Ultrastructurally, rhomboid renin protogranules are noted in the cytoplasm. Immunohistochemically, the tumor cells are positive for renin, but negative for epithelial markers. The candidates of differential diagnosis are oncocytoma, epithelioid angiomyolipoma, renal cell carcinoma, and mesenchymal tumors such as glomus tumor, solitary fibrous tumor, and hemangiopericytoma. Pathological diagnosis is usually straightforward, because of characteristic clinical presentation [4]. In cases of nonfunctioning JGCT, immunohistochemistry is helpful for differential diagnosis. Because of negative reaction with epithelial markers (cytokeratin, epithelial membrane antigen), RCC and oncocytoma are excluded. Unlike epithelioid angiomyolipoma, JGCT is not reactive with HMB45 antibody raised against melanosome-associated antibody. Although hemangiopericytomatous proliferating pattern is common with hemangiopericytoma and solitary fibrous tumor, differential diagnosis can be performed by detection of intracytoplasmic rennin with immunohistochemistry and/or electron microscopy [3]. 

Because JGCT is mostly benign with an exceptional case causing metastasis [10], nephron-sparing surgery, such as partial nephrectomy, should be considered.

Although the classical triad of JGCT is poorly controlled hypertension, elevated renin level, and renal mass, some cases had been reported which lacked hypertension and/or hypokalemia [4–6]. According to presence or absence of the symptoms, JGCT is subdivided into three groups [6]. First, the typical variant, accounting for the majority, presents elevated plasma renin activity and secondary hyperaldosteronism causing hypokalemia and hypertension. Second is the atypical variant showing hypertension but normal potassium level, which is common next to the typical variant. The third is the nonfunctioning variant, characterized by normal blood pressure and normal potassium level. Nonfunctioning variant is the rarest and is thought to produce inactive rennin [5]. Our case is classified into nonfunctioning variant. Due to lack of evidence of excessive renin secretion, we failed to consider the possibility of JGCT preoperatively. Preoperative imaging analysis on predictable histological types was limited because characteristics of renal tumors except typical clear cell renal cell carcinoma as seen in different imaging techniques have not been precisely clarified due to the limited number of cases. Enhanced pattern on CT of this case was not typical of clear cell renal cell carcinoma. The possibility of other type of RCC or low fat AML was considered to be discussed, because the incidence of AML in a Japanese young female is relatively high. Biopsy was an option of preoperative diagnosis. We explained to the patient and her family that the tumor was possibly not a renal cell carcinoma but a benign tumor such as AML, and they chose an operation instead of a true cut biopsy on FNA cytology. 

In cases of renal tumors especially in young females, even though no evidence of hyperreninism, JGCT should be included in the candidates of differential diagnosis, and excessive surgical treatment should be avoided to preserve the renal function and not to lose the kidney.
